# Residual tricuspid regurgitation after tricuspid valve interventions: What do we know?

**DOI:** 10.1002/ejhf.3675

**Published:** 2025-05-05

**Authors:** Lukas Stolz, Charles Davidson, Volker Rudolph, Jörg Hausleiter

**Affiliations:** ^1^ Medizinische Klinik und Poliklinik I, LMU Klinikum, LMU München Munich Germany; ^2^ German Center for Cardiovascular Research (DZHK), partner site Munich Heart Alliance Munich Germany; ^3^ Northwestern University Feinberg School of Medicine, Bluhm Cardiovascular Institute Chicago IL USA; ^4^ General and Interventional Cardiology/Angiology, Heart and Diabetes Center NRW, University Hospital of the Ruhr‐University Bochum, Medical Faculty OWL Bad Oeynhausen Germany

## Introduction

Tricuspid regurgitation (TR) is a major health burden being associated with substantial morbidity, mortality and quality of life (QoL) impairment.^1^ Advancements in transcatheter tricuspid valve interventions (TTVI) have significantly changed the landscape of tricuspid valve (TV) disease enabling treatment of elderly patients at advanced or prohibitive surgical risk. TV transcatheter edge‐to‐edge repair (T‐TEER) is the most commonly used treatment technique which has shown QoL improvement beyond medical treatment in randomized controlled settings.^2^, ^3^ Due to advancements in transcatheter TV replacement (TTVR)^4^, ^5^, ^6^, ^7^, ^8^ which is able to almost completely eliminate TR, a debate has arisen about how much TR reduction is desirable, safe and necessary.

Over the past months and years, the body of evidence on this question has become more substantial. In this viewpoint, we therefore present our perspective on the topic based on the data available today.

## Tricuspid regurgitation reduction in clinical trials and real‐world practice

The TRILUMINATE trial reports 30‐day TR reduction to ≤2+ in 90% of patients and ≤1+ in 52% of patients in the interventional study arm.^3^ In the Tri.FR study, TR was reduced to ≤2+ in 79% of patients and ≤1+ in 38% of patients.^2^ Core‐laboratory supervised real‐world registries for the TriClip and PASCAL device published similar rates of TR reduction (*Table* *1*). In the PASTE registry (T‐TEER using the PASCAL device), TR was reduced to ≤2+ in 87% and to ≤1+ in 55% of patients at discharge.^9^ The bRIGHT registry (T‐TEER using the TriClip device) reported TR reduction to ≤2+ in 85% and to ≤1+ in 53% of patients at 30‐day follow‐up.^10^ The large, multicentre, real‐world EuroTR registry, which includes more advanced TR and comorbid patients compared to the above‐mentioned studies and registries, published slightly lower TR reduction rates. At discharge TR was reduced to ≤2+ in 82.4% and to ≤1+ in 42.4% of patients.^11^ In contrast, TTVR using the Evoque valve system (Edwards Lifesciences) was associated with TR reduction to ≤1+ in almost all patients (98.1%).^12^

**Table 1:** Tricuspid regurgitation rates at discharge or 30‐day follow‐up in currently available transcatheter tricuspid valve intervention studies and registries

TR at discharge or 30 days	TRILUMINATE^3^	Tri.Fr^2^	PASTE^9^	bRIGHT^10^	EuroTR^11^	TRISCEND II^12^
≤1+	52%	38%^a^	55%	53%	42.4%	98.1%
2+	38%	41%^a^	32%	32%	40.0%	1.9%
≥3+	10%	21%^a^	13%	15%	17.6%	0.0%

TR, tricuspid regurgitation.

^a^ Estimated values derived from supplemental charts of the Tri.Fr study.

An almost complete elimination of TR after TTVR comes at the cost of a higher overall complication rate compared to T‐TEER. The TRISCEND II study identified conduction disturbances requiring pacemaker implantation (24.7% of pacemaker‐naive TTVR patients) and bleeding complications (15.4% of TTVR patients) as the most common adverse events. In contrast, T‐TEER is associated with extremely low procedural complication rates as demonstrated by TRILUMINATE and Tri.Fr.

## Prognostic implications of residual tricuspid regurgitation

### Survival and survival free from heart failure hospitalization

Historically, T‐TEER has been considered successful in case of TR reduction to moderate or less (≤2+). Several registries have reported a significant impact of successful TR reduction on survival and survival free from heart failure hospitalization (HFH).^10^, ^11^, ^13^ In a multivariate Cox regression model derived from the EuroTR registry, residual TR (≤2+) was a strong and independent predictor for a lower 2‐year mortality after T‐TEER (hazard ratio 0.5, 95% confidence interval 0.3–0.77, *p* < 0.001).^11^ Similar results have been reported in the bRIGHT registry where significant TR reduction to ≤2+ was associated with a reduced 1‐year mortality risk (odds ratio 0.32, 95% confidence interval 0.19–0.56, *p* < 0.001).^14^ Interestingly, no survival differences were observed when comparing residual TR 1+ versus residual TR 2+. This has been reported consistently in EuroTR, PASTE, bRIGHT and the TRIGISTRY.^9^, ^11^, ^13^, ^14^ However, it should be noted that the majority of those studies only examined a follow‐up period up to 2 years. Whether a comparison between residual TR 1+ versus 2+ could have a long‐term prognostic impact on survival or HFH cannot be excluded based on the currently available short‐ and mid‐term outcome data. Beyond that, the TRIGISTRY divided patients with moderate residual TR into those with mild‐to‐moderate and moderate‐to‐severe. Two‐year survival rates of patients with mild‐to‐moderate and mild residual TR were comparable, as well as the ones of patients with moderate‐to‐severe and severe residual TR.^13^ This once again emphasizes the challenges of TR quantification especially after TEER with one or several devices in place.

Studies not only focused on TR reduction to a certain level but also analysed the impact of the absolute degree of TR reduction in survival outcomes. Despite not reaching clinical significance, better absolute TR reduction seemed to be linked with better survival in the EuroTR registry.^11^

### Symptomatic outcomes, functional capacity and quality of life improvement

Given that prospective randomized controlled clinical trials in the field of TTVI have so far demonstrated an impact only on QoL, but not on hard clinical endpoints such as survival or HFH, it is important to also examine the impact of TR reduction on these symptomatic endpoints. The current literature suggests that the majority of symptomatic endpoints are significantly correlated with the absolute degree of TR reduction. In EuroTR, the proportion of patients with an improvement of New York Heart Association functional class by one or two degrees increased with the degree of TR reduction (TR reduction by one grade: 51%, by two grades 60%, by three grades 63%).^11^ Within the TRILUMINATE trial, QoL as expressed by the Kansas City Cardiomyopathy Questionnaire (KCCQ) did not improve from baseline to 1‐year follow‐up in patients with worsening or unchanged TR. On the other hand, in patients with one grade TR reduction it improved by a mean of 10 points and in case of two or more grades TR reduction by 17 points.^5^, ^15^ Equivalent results were reported for 6‐min walking distance (6MWD). Worsening or unchanged TR was associated with a worsening in 6MWD while functional capacity improved in parallel with TR reduction.^13^ Similarly, a sub‐analysis from the TRISCEND II study confirmed those findings.^5^ Patients with massive or torrential TR at baseline presented with more pronounced KCCQ improvement compared to those with ‘only’ severe TR at baseline and thus a smaller absolute degree of TR reduction.^5^, ^7^

### Right ventricular reverse remodelling

Transcatheter TV intervention is known to be associated with significant right ventricular reverse remodelling (RVRR).^16^, ^17^ In patients undergoing T‐TEER, RVRR is a biphasic process involving early right ventricular volume unloading (reduction in right ventricular end‐diastolic volumes [RVEDV]) and later structural RVRR (reduction in right ventricular end‐systolic volumes [RVESV]).^18^ As recently shown in a multicentre study using three‐dimensional echocardiography and cardiac magnetic resonance imaging, both, right ventricular volume unloading and structural RVRR significantly correlate with the degree of TR reduction.^19^ In patients with procedural TR reduction to ≤1+, RVEDV and RVESV were reduced by 18% and 8%, respectively, compared to 10% and 2% in patients with residual TR 2+. In patients with remaining severe or greater TR, RVEDV and RVESV even increased by 4% and 6%, respectively.^19^

Patient selection for TTVI remains a major challenge and warrants a multiparametric approach taking into consideration multiple factors. If sustainable TR reduction by T‐TEER seems likely based on our current knowledge, the latter should be considered as primary treatment approach due to its excellent safety profile. Patients clearly being at risk of relevant residual TR after T‐TEER should undergo screening for TTVR as a potential alternative treatment technique.

It can be summarized that, although there is no difference in 1‐ or 2‐year survival between patients with residual TR 1+ versus 2+, the degree of TR reduction appears to have an important ‘dose–response‐like’ impact on symptomatic outcomes and RVRR following TTVI. The association between the degree of TR reduction and symptomatic as well as QoL benefits further suggests that, despite the lack of sham‐controlled designs in previous TTVI studies, a causal relationship between TR reduction and outcome improvement may exist.

## Predicting residual tricuspid regurgitation after tricuspid transcatheter edge‐to‐edge repair

**Figure 1:**
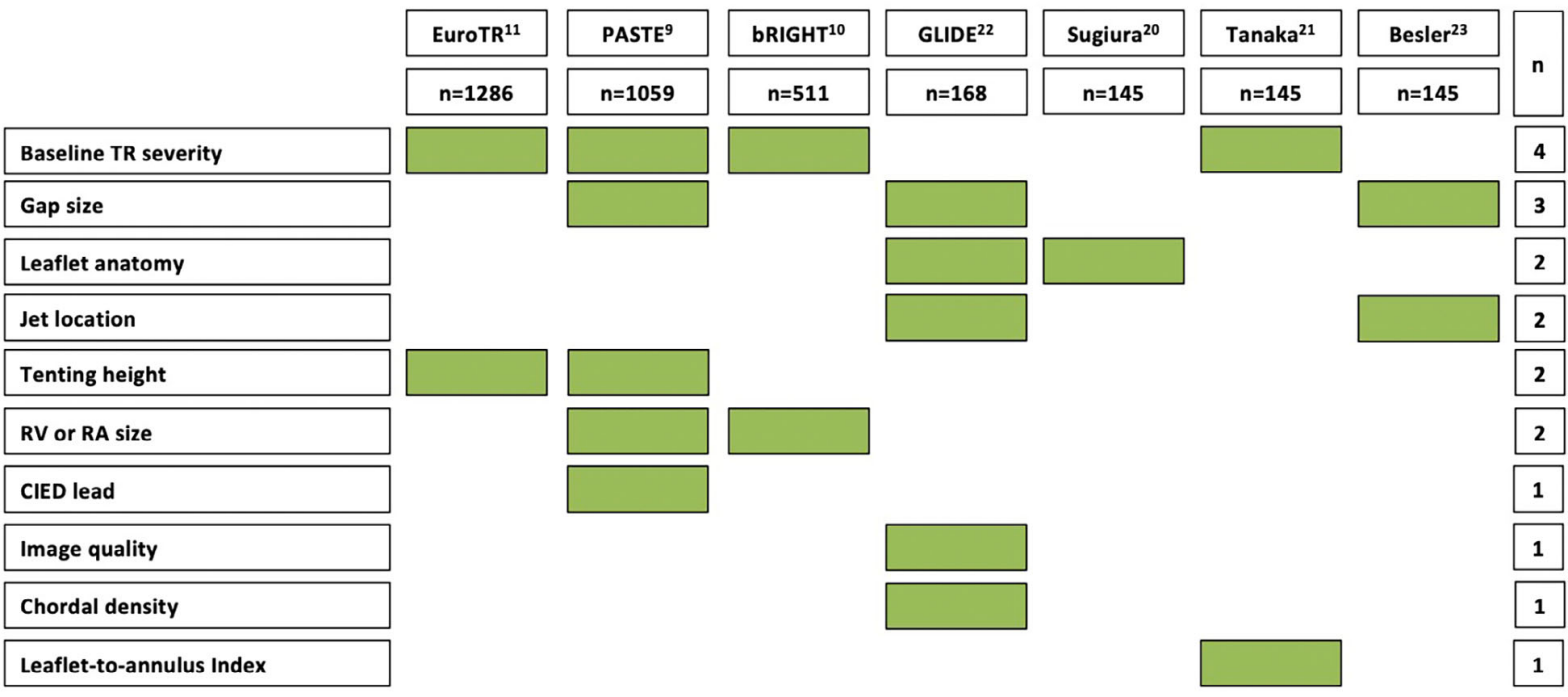
Currently known predictors for relevant residual tricuspid regurgitation (TR) after tricuspid transcatheter edge‐to‐edge repair. CIED, cardiac implantable electronic device; RA, right atrium; RV, right ventricle; TR, tricuspid regurgitation. Green boxes indicate studies that identified the respective parameter as predictor for relevant residual TR.

Given the results described so far, predicting procedural outcomes after T‐TEER as effectively as possible seems crucial for patient selection and device allocation within the heart team. A number of studies have identified several parameters associated with an increased risk of residual TR after T‐TEER. Some predictors have been identified in multiple studies, while others have been found only in individual smaller single‐centre analyses. *Figure* *1* provides an overview of the predictors identified so far for residual TR after T‐TEER. Overall, the risk of significant residual TR seems to accumulate with increasing baseline severity of TR, and consequently a larger gap size and more pronounced tenting. Beyond that, single studies identified the presence of a trans‐tricuspid cardiac implantable electronic device lead,^9^ TV anatomy,^20^ TV leaflet‐to‐annulus ratio^21^ to be associated with residual TR after T‐TEER. An extremely challenging aspect, however, remains the prediction of the procedural outcomes at the individual patient level. The recently published GLIDE score for the first time provides a tool for prediction of post‐procedural TR reduction by ≥2 grades and TR grade moderate or less. Being derived from 168 T‐TEER patients and externally validated in 126 patients from independent institutions, the score comprises five echocardiographic parameters: septo‐lateral and antero‐posterior coaptation gap, leaflet morphology, chordal structure density, en face TR jet morphology and TR jet location and image quality.^22^ With an area under the curve for procedural success of 0.77 (95% confidence interval 0.69–0.86) in the validation cohort, the GLIDE score is the only available scoring system for procedural outcomes after T‐TEER. A recent multicentre evaluation confirmed the predictive accuracy for obtaining a residual TR ≤1+ after T‐TEER in patients with a low GLIDE score. Whether further improved scores or artificial intelligence, potentially also through direct image analysis, will enable even better predictions of procedural outcomes remains to be seen. Until then, a detailed discussion within the interdisciplinary heart team is essential, especially considering other available therapeutic options for patients with severe TR beyond TEER. Especially in patients with higher GLIDE score values and a significant probability of relevant residual TR, alternative imaging techniques including intracardiac echocardiography for improving T‐TEER outcomes or TTVR needs to be discussed as a primary treatment approach.

Another major challenge is the quantification of TR after TEER. From the field of mitral valve interventions, it is well known that the proximal isovelocity surface area method is limited in its accuracy after TEER. This is because the method assumes that the regurgitant flow passes through a symmetrical and focused opening of the valve. Explicit recommendations on this have not yet been systematically published or integrated into guidelines. Whether cardiac magnetic resonance could potentially facilitate quantification of residual TR remains to be seen and currently is not part of clinical routine.

## Conclusions

Although no survival differences after 1 or 2 years have been observed for patients with mild versus moderate residual TR, the amount of TR reduction which is usually more pronounced after TTVR compared to TEER is associated with the degree of symptomatic outcome, QoL improvement, and more pronounced RVRR. Balancing the benefits and risks, the goal of any TTVI should be to achieve the maximum possible TR reduction.
